# *Escherichia coli* as Reservoir for Macrolide Resistance Genes

**DOI:** 10.3201/eid1510.090696

**Published:** 2009-10

**Authors:** Minh Chau Phuc Nguyen, Paul-Louis Woerther, Mathilde Bouvet, Antoine Andremont, Roland Leclercq, Annie Canu

**Affiliations:** Université de Caen, Caen, France (M.C.P. Nguyen, M. Bouvet, R. Leclercq, A. Canu); University Paris-Diderot Medical School, Paris, France (P.-L. Woerther, A. Andremont)

**Keywords:** Antimicrobial resistance, shigellosis, azithromycin, erythromycin, Escherichia coli, bacteria, dispatch

## Abstract

The plasmid-borne *mph*(A) gene that confers resistance to azithromycin and has recently emerged in *Shigella sonnei* is present in multidrug- and non–multidrug-resistant *Escherichia coli* isolates from 4 continents. Further spread of *mph*(A) to *Shigella* and *Salmonella* spp. may be expected.

Macrolides have been regarded for many decades as having good activity and safety for the treatment of infections caused by gram-positive cocci. In general, macrolides show modest potency against *Enterobacteriaceae*. Most *Shigella* and *Salmonella* spp. pathogens display MICs of azithromycin, a macrolide antimicrobial drug, ranging from 2 mg/L to 8 mg/L (*1*). Despite these relatively high MICs, azithromycin is an attractive option for several reasons. It can be given once a day and attains high intracellular concentrations and sufficient concentrations in the colon of patients to inhibit *Shigella* and *Salmonella* spp. Azithromycin is recommended by the American Academy of Pediatrics for treatment of shigellosis in children (*2*) and by the World Health Organization as a second-line treatment for adults (*3*). It has also been proposed for short-course treatment of typhoid fever (*4*).

We recently reported an outbreak of shigellosis in Paris, France; failure of azithromycin treatment was related to emergence of plasmid-mediated resistance to macrolides (*5*). Resistance was related to the expression of a macrolide 2′ phosphotransferase encoded by the *mph*(A) gene. Because shigellosis remains a common gastrointestinal disease in both developing and industrialized countries, emergence of macrolide resistance may have major public health consequences.

Since the early reports by Ochiai (*6*) and Akiba (*7*) at the end of the 1950s, plasmid-mediated transfer of resistance genes between *Escherichia coli* and *Shigella* spp. has been reported in several instances (*8*). Therefore, we hypothesized that *E. coli* might constitute a major reservoir for macrolide resistance genes that could be subsequently transferred to *Shigella sonnei*.

Acquired resistance to macrolides may result from a variety of mechanisms of resistance, several of which have already been reported in *Enterobacteriaceae* (*9*,*10*). These mechanisms include target site modification by methylases encoded by *erm* genes, in particular *erm*(A), *erm*(B), and *erm*(C). Macrolides may be inactivated by modifying enzymes first reported in *Enterobacteriaceae* (*11*,*12*), e.g., esterases encoded by *ere*(A) or *ere*(B) genes or phosphotransferases encoded by *mph*(A), *mph*(B), and *mph*(D) genes. The third mechanism is acquisition of efflux pumps, *mef*(A) and *msr*(A), that have been found essentially in gram-positive organisms, although *mef*(A) has been identified in gram-negative organisms (*10*). All of these genes confer full cross-resistance between erythromycin and azithromycin (*9*). We aimed to assess the prevalence of acquired resistance to macrolides in commensal and clinical isolates of *E. coli* from various geographic origins and to characterize the mechanisms underlying E. coli resistance to macrolides.

## The Study

A total of 190 *E. coli* isolates were collected from 5 countries on 4 continents. Some of these isolates were obtained from populations exposed to low antimicrobial selective pressure; 45 commensal isolates were from feces of healthy Wayampi Amerindians in French Guiana, 20 from feces of children living in a remote village of Senegal, and 49 from feces of healthy nurses working in a hospital in Paris. Other isolates were obtained from populations having received multiple antimicrobial drug treatments; 29 isolates were from feces of children from Niger hosted in a center for nutritional rehabilitation, and 47 isolates were producers of extended-spectrum β-lactamase (ESBL) obtained from various clinical samples in hospitalized patients in Vietnam (n = 37) and France (Hospital of Caen) (n = 10).

Susceptibility to 16 antimicrobial drugs was determined by the disk-diffusion method. MICs of erythromycin were determined by the agar dilution technique, and ESBLs were detected by the double-disk synergy test, as recommended by the French Society for Microbiology (www.sfm.asso.fr).

*E. coli* isolates from French Guiana, Senegal, and Paris were susceptible to quinolones, gentamicin, and third-generation cephalosporins. Resistance to amoxicillin-ticarcillin (by penicillinase production) was detected for 22.2%, 20.4%, and 40.0% of the isolates obtained from nurses in Guiana, Paris, and Senegal, respectively. Coresistance to amoxicillin and cotrimoxazole was found for 13%, 14%, and 35% of isolates, respectively.

Multidrug-resistant isolates were commonly obtained from Niger natives; 34.4% were resistant to both cefotaxime (mostly by ESBL production) and ciprofloxacin, and 58.6% to gentamicin. ESBL producers from Vietnam and Caen hospital displayed resistance to ciprofloxacin for 86.5% and 60.0% and resistance to gentamicin for 86.4% and 50.0% of isolates, respectively. MICs of erythromycin ranged from 16 mg/L to >1,024 mg/L (Table 1; Figure). Distribution of MICs of erythromycin was bimodal; mode = 64 mg/L for 1 population with low MICs and >1,024 mg/L for the other population with high MICs (Figure).

**Table 1:** MICs of erythromycin and distribution of macrolide resistance genes among 190 *Escherichia coli* isolates from 5 countries*

*E. coli* origin (no. isolates)	MIC of erythromycin, mg/L				Gene, no. (%)†		
	Range	MIC_50_	MIC_90_		*erm*(B)	*mph*(A)	*mph*(B)
French Guiana, Amerindians (45)	32–1,024	64	128		0	1 (2)	0
Senegal, remote village (20)	64–128	128	128		0	0	0
Niger, children (29)	64–>1,024	256	>1,024		0	9 (31)	0
France, healthy nurses (49)	16–256	64	128		0	2 (4)	1 (2)
France, ESBL isolates (10)	64–1,024	128	1,024		0	3 (30)	1 (10)
Vietnam, ESBL isolates (37)	32–>1,024	512	>1,024		5 (13.5)	19 (51)	0
France, hospital isolates resistant to ampicillin and cotrimoxazole (100)	32–>1,024	64	>1,024		1 (1)	13 (13)	0

*ESBL, extended-spectrum β-lactamase; MIC_50_, MIC at which 50% of isolates are inhibited; MIC_90_, MIC at which 90% of isolates are inhibited. †No isolate contained the *erm*(A), *erm*(C), *ere*(A), *ere*(B), *msr*(A), or *mef*(A) genes.

**Figure:**
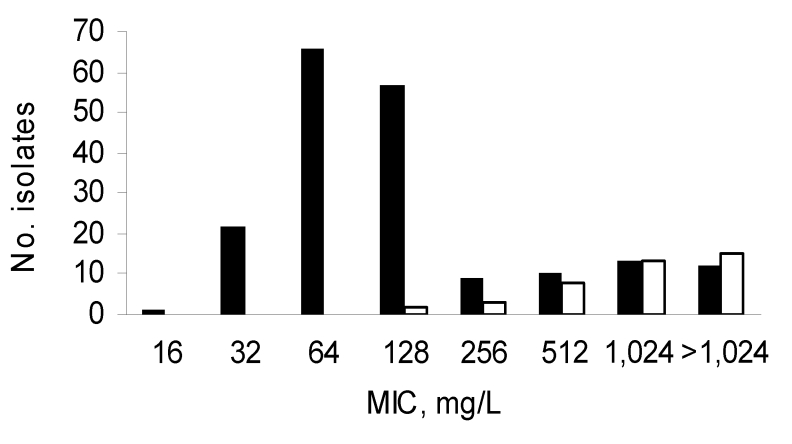
Distribution of MICs of erythromycin for *Escherichia coli* isolates according to the presence of genes resistant to macrolides. MIC distribution is shown for all strains (black bars). Solid white bars indicate strains containing a macrolide resistance gene: *erm*(B), *mph*(A), or *mph*(B). Some isolates may contain 2 genes resistant to macrolides.

MICs differed according to the origin of the isolates. Multiple resistance was associated with MICs of erythromycin >256 mg/L with 1 exception: an isolate from Guiana was resistant only to amoxicillin and cotrimoxazole (MIC of erythromycin, 1,024 mg/L).

We screened for macrolide resistance genes by using oligonucleotide primers and PCR conditions (Table 2). PCR reactions were performed as follows: an initial denaturation step (95^o^C, 3 min) followed by 30 cycles consisting of denaturation (95^o^C, 30 s), annealing at a temperature depending on the primers used (30 s), elongation (72^o^C, 30 s) and a final extension step (72^o^C, 10 min). Positive and negative controls were included in each run.

**Table 2:** Oligonucleotide primers used for detection of *Escherichia coli* macrolide resistance genes

Target gene	Primer	Sequence, 5′ → 3′	Product size, bp	Annealing temperature, ^o^C
*mph*(A)	mphAF	GTGAGGAGGAGCTTCGCGAG	403	60
	mphAR	TGCCGCAGGACTCGGAGGTC		
*mph*(B)	mphBF	GATATTAAACAAGTAATCAGAATAG	494	58
	mphBR	GCTCTTACTGCATCCATACG		
*erm*(A)	ermAF	TCTAAAAAGCATGTAAAAGAAA	533	52
	ermAR	CGATACTTTTTGTAGTCCTTC		
*erm*(B)	ermBF	GAAAAAGTACTCAACCAAATA	639	45
	ermBR	AATTTAAGTACCGTTACT		
*erm*(C)	ermCF	TCAAAACATAATATAGATAAA	642	45
	ermCR	GCTAATATTGTTTAAATCGTCAAT		
*ere*(A)	ereAF	GCCGGTGCTCATGAACTTGAG	420	60
	ereAR	CGACTCTATTCGATCAGAGGC		
*ere*(B)	ereBF	TTGGAGATACCCAGATTGTAG	537	55
	ereBR	GAGCCATAGCTTCAACGC		
*mef*(A)	mefAF	AGTATCATTAATCACTAGTGC	345	54
	mefAR	TTCTTCTGGTACTAAAAGTGG		
*msr*(A)	msrAF	GCACTTATTGGGGGTAATGG	384	58
	msrAR	GTCTATAAGTGCTCTATCGTG		

The *mph*(A) gene was commonly present in 34 isolates (MICs 256 mg/L to >1,024 mg/L). The gene was mostly detected in isolates resistant to cefotaxime (27 isolates) but also in 4 (21%) of 19 isolates resistant to only amoxicillin and cotrimoxazole in different countries. To confirm this latter association, we searched for the *mph*(A) gene in 100 clinical isolates of *E. coli* from the Caen hospital coresistant to amoxicillin and cotrimoxazole but susceptible to cefotaxime, which is a common phenotype present in ≈25% of *E. coli* isolates from this hospital. The gene was detected in 13 isolates (MIC >256 mg/L), confirming the presence of the gene in non–multidrug-resistant *E. coli* (Table 1). In a previous study on the distribution of 7 macrolide resistance genes in gram-negative isolates from the urine and oral cavity of healthy children in Portugal, Ojo et al. detected the *mph*(A) gene in 15 of 26 studied *E. coli* isolates (*10*). However, the profile of resistance to other antimicrobial drugs was not determined.

The other macrolide resistance genes were more scarce. The *erm*(B) gene was detected in 2 isolates (MICs >1,024 mg/L) and *mph*(B) in 2 others (MICs 128 mg/L). In 4 isolates (MICs >1,024 mg/L), both *mph*(A) and *erm*(B) were amplified. The 6 other genes, *erm*(A), *erm*(C), *ere*(A), *ere*(B), *mef*(A), and *msr*(A), were not detected. In 6 isolates with MICs of erythromycin equal to 256 mg/L and 2 with MICs of erythromycin equal to 512 mg/L, no resistance gene could be amplified, suggesting the presence of other macrolide resistance determinants. Distribution of the resistance genes *mph*(A), *erm*(B), and *mph*(B) is shown in Table 1 and in the Figure.

## Conclusions

The plasmid-borne *mph*(A) gene detected in *S. sonnei* resistant to azithromycin was the most common macrolide resistance gene detected in *E. coli* collected in 5 countries on 4 continents. The gene was mostly detected in isolates from patients who had received antimicrobial drugs or had been hospitalized, in particular in ESBL producers, but was also detected in *E. coli* isolates coresistant to amoxicillin and cotrimoxazole, which are common worldwide. Because *E. coli* and *Shigella* spp. are phylogenetically closely related species that easily exchange plasmids, further dissemination of resistance to macrolides in the latter species may be predicted. Also, plasmid-mediated resistance to macrolides may emerge in *Salmonella* spp., which is also a target of azithromycin.
